# Assessment of the appropriateness of stress ulcer prophylaxis use and its determinants among admitted surgical patients at Debre Berhan University Hakim Gizaw Hospital, Ethiopia. A hospital-based cross-sectional study

**DOI:** 10.3389/fmed.2024.1345144

**Published:** 2024-04-05

**Authors:** Abate Wondesen Tsige, Dessale Abate Beyene, Yehualashet Teshome Wondmkun, Bedilu Linger Endalifer, Habtemariam Alekaw Habteweld, Fissha Assegidew Gebretadik, Aregahegn Adafir Gebeyehu, Belayneh Abebaw Azene, Misganaw Abebaw Alamneh, Daniel Zebene Tesfaye, Misganaw Aynalem Fered, Mandefro Teje Girma, Melkamu Belayneh Mekonen, Tigist Yazezew Dessie, Siraye Genzeb Ayele

**Affiliations:** ^1^Department of Pharmacy, College of Health Sciences, Debre Berhan University, Debre Berhan, Ethiopia; ^2^Department of Pharmacy, College of Health Sciences, Debre Berhan Health Science College, Debre Berhan, Ethiopia; ^3^Department of Pharmacy, Debre Berhan University Hakim Gizaw Hospital, Debre Berhan, Ethiopia; ^4^Department of Midwifery, School of Nursing and Midwifery, College of Health Sciences, Addis Ababa University, Addis Ababa, Ethiopia

**Keywords:** stress ulcer prophylaxis, stress ulcer, American Society of Health-System Pharmacists, surgical patients, Ethiopia

## Abstract

**Introduction:**

Pharmacological stress ulcer prophylaxis (SUP) has been recommended for many years to reduce the risk of clinically significant upper gastrointestinal (GI) bleeding caused by stress ulcers (SUs). Stress-related ulcer bleeding in surgical patients significantly increases morbidity and mortality. Therefore, preventing stress-induced hemorrhage is the most appropriate measure for patients who are at increased risk. However, the inappropriate use of SUP has increased in recent years, and its use in Ethiopian surgical patients has not been well studied.

**Objective:**

The aim of this study was to assess the appropriateness of SUP use and its determinants among admitted surgical patients at Debre Berhan University Hakim Gizaw Hospital (DBUHGH), Ethiopia.

**Methods:**

We randomly selected 230 patients from the whole cross-sectional group of all surgical patients at DBUHGH from 1 February to 30 June 2023. The risk of stress ulcer (SU) development was assessed using the modified American Society of Health-System Pharmacists (ASHP) guidelines. For data analysis, we used SPSS version 25.

**Results:**

The mean age of study participants was 47.2 years (SD ± 20.4), and out of the total of 230, 130 (56.5%) were women. Approximately 66% of study participants took inappropriate SUP based on ASHP guidelines criteria. The most commonly used drug class for SUP was histamine-2 receptor blockers 115 (50%). Study participants who have a Charlson Comorbidity Index Score of moderate and GI bleeding have been significantly associated with the inappropriate use of SUP.

**Conclusion:**

In our study, inappropriate SUP use was common in the surgical ward of DBUHGH. This may be an area that requires further and more focused working together among clinical pharmacists and medical professionals in an institution-specific SUP protocol that aids clinicians in identifying appropriate candidates for SUP medication.

## Introduction

Stress ulcer (SU) is a type of hemorrhagic gastritis that can occur in critically ill patients who have experienced a moderate to severe physiological stress event (1–3). The regional bleeding associated with SUs that are accompanied by mucosal obstruction also affects the upper gastrointestinal (GI) system (4). The development of this condition is influenced by several factors, such as increased acid production, changes in the gastric mucosa’s epithelial turnover, and abnormal secretion of mucus and bicarbonate (5). Stress-related mucosal damage (SRMD) is classified into two distinct categories: broad, surface epithelial damage and deep, localized SUs that penetrate the sub-mucosa. These ulcers typically affect the GI system and fundal regions of the intestines (6, 7).

Mucosal damage and ulceration are significantly influenced by decreased blood circulation, mucosal ischemia, inadequate perfusion, and circulatory disturbances (8, 9). In addition, a variety of components, including hyper-secretion of acids, alterations in routine defense mechanisms including mucosal and bicarbonate fluids, the release of arachidonic acid, cytokines, and free radicals from oxygen, and ischemia of the GI system, contribute to the development of SUs (9–11). This damage can develop immediately (usually only 24 h after ingestion) or gradually (throughout more than 10–14 days) (10).

In critically ill people, stress-related ulcer bleeding significantly increases morbidity and mortality (12), and the mortality rate ranges from 37 to 77% (13–15). Bleeding from the upper GI tract is one of the most common symptoms of stress-related ulceration (12). Prevention of stress-induced hemorrhage is the most appropriate measure for patients who are at increased risk for SRMD (9, 11, 16). Although excessive acidity is not the main cause of SRMD, regulation of acid release seems to be preventive against bleeding episodes in vulnerable individuals (17, 18). The use of pharmacological stress ulcer prophylactics (SUP) has been encouraged for many years to reduce the risk of clinically serious upper GI bleeding caused by SUs (19, 20).

Proton pump inhibitors (PPIs), histamine-2 receptor blockers (H2RBs), and sucralfate are available as prophylactic alternatives. The choice of the type of prophylaxis can be influenced by a variety of parameters, including the presence of risk factors, the possibility of hospital-acquired pneumonia, and cost (19, 21–23).

In terms of reducing the risk of clinically significant bleeding from the GI tract, a meta-analysis reported that PPIs are significantly more beneficial than H2RB, sucralfate, and placebo (24). However, the scientific studies and recommendations for the critically ill group recommend the administration of PPIs or H2RB as SUP (25, 26). In the literature, inappropriate SUP medication use (drugs given without indication) in surgery patients was common (27). The study conducted by Maz, Chen, and Chu et al. concluded that 22–97% of SUP was administered to surgical inpatients without a clear indication (27–29).

There are no prior studies to evaluate the appropriateness of SUP among surgical patients admitted to DBUHGH. Moreover, there is a limited study examining the suitability of SUP among admitted surgical patients in Ethiopia. Therefore, the objective of this study was to assess the appropriateness of SUP use and its determinants among surgical patients admitted to DBUHGH. The findings of the study will help researchers and decision-makers thoroughly understand how clinicians use SUP and offer practical solutions for SUP management.

## Materials and methods

The STROBE checklist was followed for this cross-sectional study.

### Study area, design, and period

A hospital-based study was conducted in DBUHGH from 1 February to 30 June 2023, among surgical ward admitted patients. Debre Berhan is the administrative city of North Shoa Zone, Amhara regional state, Ethiopia (30, 31–34).

### Population

All surgical ward admitted patients in DBUHGH during the study period were the source population, while patients who fulfilled our inclusion criteria, who took up SUP, and who had been admitted during the study period were the study population.

### Eligibility criteria

We randomly selected 230 patients from the whole cross-sectional group of all surgical patients aged ≥18 years who underwent surgical operations in the surgical department, had at least a hospital stay length of 2 days, had risk factors for stress-induced ulcers according to ASHP guidelines criteria, and had taken acid-suppressive therapy. Whereas, study participants who had a history of peptic ulcers, acid-suppressive medication prescriptions for the treatment of GI diseases such as ulcers, esophagitis, dyspepsia, gastroesophageal reflux disease, or epigastric pain within 1 month before admission, or a new onset of GI disease during hospitalization confirmed by endoscopy, were excluded from the study.

### Variables

Appropriateness of SUP was the dependent variable, while study participant demographics (occupation, age, social drug use, educational status, living status, and marital status) and clinical characteristics (number of comorbidities, type of diagnosis, type of acid suppressant therapy used, duration of hospital admission, presence of hospital admission history, and concomitant drug use) were predictor variables.

### Sample size determination and sampling technique

The sample size was computed using a single population proportion formula. Considering the 50% prevalence of SUP in Ethiopia (35, 36), since there were no previous studies performed in the current study area. Using a margin of error of 5% at a 95% confidence level resulted in 384.

The expected number of individuals in the source population during the study period (N), based on the average number of patients admitted to the surgical ward who received surgical services within the total 6-month study period, was 463. The corrected sample size, using the following correction formula, was 209.9 ≈ 209,

Corrected sample size = $\frac{\;n\times N}{n+N\text{.}}$

Then 10% contingency of non-response rate is added on 209; 209 × 10% = 21

209 + contingency = Nf = 230

A simple random selection was employed to select study participants from the electronic medical record (EMR) system of the DBUHGH surgical ward who met the eligibility criteria.

### Data quality assurance, collection instrument, and collection process

The data were collected using pre-tested structured data abstraction tools from the EMR of surgical ward admitted patients, which contains all relevant variables based on the objectives of the study. The first part of the structured data abstraction tool contained socio-demographic data, and the second part was the clinical characteristics of the study participants.

### Assessment of SUP appropriateness

The appropriateness of SUP was determined using modified American Society of Health-System Pharmacists (ASHP) guidelines with various SUP protocols summarized in Table 1 (10, 37–39). The appropriateness of SUP was identified by clinical pharmacists who were trained on the study protocol in a special workshop that was held by the principal investigator of the study. The inappropriateness of SUP was identified from the collected data using the above guidelines, reviewed literature, drugs.com, Micromedex, and up-to-date resources. The identified inappropriate SUPs were recorded using the data abstraction format, which is taken from ASHP guidelines.

**Table 1:** Major and minor risk factors for stress ulcers used in our study based on ASHP guidelines.

**At least one major risk factor from the following**
Populations having general surgery
Coagulation related problem (a platelet count <50,000 or INR > 1.5 or a PTT > two times the control value)
Failure of respiration (mechanical ventilation greater than 48 h)
Multiple traumas with an injury severity score greater than or equal to 16
Liver and kidney failure
Head injury with a Glasgow Coma Score of ≤10 or an inability to obey simple commands
History of gastric ulceration or bleeding during the year before admission
Thermal injury involving >35% of body surface area
**The presence of at least two of the following minor risk factor**
History of NSAIDs >3 months of use
Current high-dose NSAID therapy (ibuprofen >1,200 mg/day, naproxen >1,000 mg/day, all scheduled ketorolac regimens)
Prolong NPO status lasting >5 days with GI pathology or after major surgery
Use of heparin with the therapeutic dose
Corticosteroid therapy (>250 mg hydrocortisone or equivalent)
Sepsis
Occult or overt bleeding for ≥6 days
Use of two antiplatelet agents (i.e., clopidogrel, aspirin, cilostazol, ticagrelor, and dipyridamole)
Use of warfarin

### Data processing, analysis, and interpretations

The hand-gathered data were coded, cleaned, and imported into Epi-data 4.2.0 after being carefully validated for completeness. Data analysis made use of SPSS version 25.0. To determine the relationship between the occurrence of inappropriate SUP use and independent variables, binary logistic regression analysis was used. The multivariable binary logistic regression analysis was conducted to identify potential determinants of the inappropriateness of SUP, and all factors having a *p*-value of 0.2 in the univariable binary logistic regression analysis were included. Statistical significance was defined as a *p*-value of 0.05.

## Results

### Socio-demographic characteristics of the patients

As shown in Table 2, a total of 230 study participants took part in this study, of which more than half, 130 (56.5%), were women. Regarding age distribution, the mean age of study participants with standard deviation was 47.2 ± 20.4 years, and most 109 (47.4%) participants were in the age group of <40 years. Majority 204 (88.7%) of study participants followed Orthodox Christian. Married 181 (78.7%) made up the largest proportion. Approximately half 129 (56.1%) of the study participants could only read and write, and 50 (21.7%) were housewives. More than half of the study participants 136 (59.1%) of them lived in the city (near the hospital).

**Table 2:** Socio-demographic characteristics of patients receiving stress ulcer prophylaxis in the surgical ward of DBUHGH.

**Variables**		**Frequency**	**Percentage**
Sex	Female	130	56.5
	Male	100	43.5
Age	<40 years	109	47.4
	40–64 years	57	24.8
	≥65 years	64	27.8
Religion	Orthodox	204	88.7
	Muslim	18	7.8
	Protestant	8	3.5
Marital Status	Single	46	20.0
	Married	181	78.7
	Divorced/widowed	3	1.3
Educational Status	Unable to read and write	26	11.3
	Able to read and write	129	56.1
	Elementary school	8	3.5
	Secondary school	10	4.3
	Diploma and above	57	24.8
Occupation	Farmer	40	17.4
	Merchant	24	10.4
	Employed	37	16.1
	Unemployed	30	13.0
	Housewife	50	21.7
	Student	17	7.4
	Retried	21	9.1
	Others*	11	4.8
Residency	Rural	94	40.9
	Urban	136	59.1

Others*; Daily worker, Garage.

### Clinical characteristics of patients taking stress-induced ulcer prophylaxis

In this study, 128 (55.7%) of the study participants have a comorbidity, with 96 (41.7%) having a Charlson comorbidity index of 1–2 (mild), as indicated in Table 3. In addition, 75 (32.6%) of the study participants were taking medication for SUP at discharge. As for the degree of prescribing SUP medications, half of 117(50.9%) of them were general practitioners.

**Table 3:** Clinical characteristics of patients taking stress ulcer prophylaxis treatment in the surgical ward of DBUHGH.

**Variables**		**Frequency**	**Percentage**
Comorbidities	Yes	128	55.7
	No	102	44.3
Social drug use	Alcohol consumption	3	1.3
	Do not use	227	98.7
Level of SUP prescribers	Intern	47	20.4
	General Practitioner	117	50.9
	Specialist	66	28.7
Charlson Comorbidity	1–2 (Mild)	96	41.7
Index Score	3–4 (Moderate)	12	5.2
	≥5 (Severe)	5	2.2
History of drug allergy	Yes	2	0.9
	No	228	99.1
Stress ulcer drugs during	Yes	75	32.6
discharge	No	155	67.4

**Figure 1:**
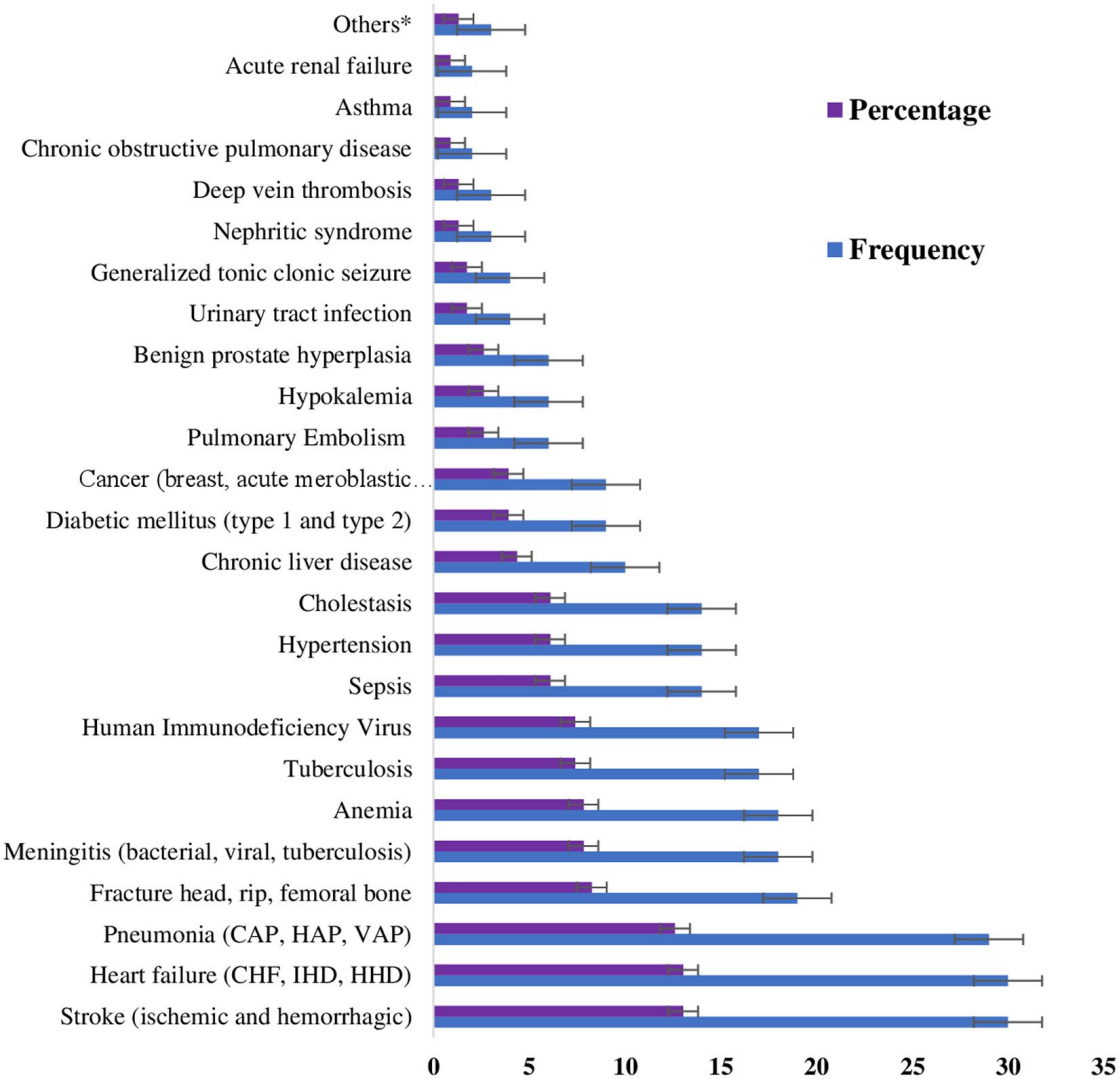
Patients’ assessments in the surgical ward of DBUHGH throughout their hospital stay. Others* Pelvic inflammatory disease, gout, and Crohn’s disease. CHF, congestive heart failure; IHD, ischemic heart disease; HHD, hypertensive heart disease; CAP, community-acquired pneumonia; HAP, hospital-acquired pneumonia; VAP, ventilator-associated pneumonia.

At Hakim Gizaw Hospital, out of all the patients who were evaluated surgically, 30 (13.04%) had suffered from stroke (ischemic and hemorrhagic), 30 (13.04%) had heart failure (congestive heart failure, ischemic heart disease, and hypertensive heart disease), followed by 29 (12.61%) with pneumonia (community-acquired, hospital-acquired, and ventilator-associated), 21 (9.13%) with gastric ulcers, and 19 (8.26%) with fractures of the head, rib, and femur, as stated in Figure 1.

### Treatment-related characteristics of study participants

As for the patients taking SUP treatment, 115 (50.0%) of the study participants were taking H2RBs, followed by 67 (29.1%) PPIs. As for the type of PPIs taken for SUP, 52 (22.6%) were taking omeprazole 40 mg daily IV. On the other hand, for H2RBs, 120 (52.2%) took cimetidine 200 mg IV BID. In addition, patients were taking other medications concurrently with SUP; in this study, 68 (29.6%) of study participants were taking NSAIDs, followed by 41 (17.8%) anticoagulants and 30 (13%) systemic corticosteroids + NSAIDs (Table 4).

**Table 4:** Treatment-related characteristics of patients receiving stress ulcer prophylaxis treatment in the surgical ward of DBUHGH.

**Variables**		**Frequency**	**Percentage**
Type of acid suppressant	Proton Pump Inhibitors	67	29.1
	Histamin-2 Receptor Blockers	115	50.0
	Histamin-2 Receptor Blockers followed by Proton pump inhibitors	48	20.9
Proton Pump Inhibitors	Omeprazole 40 mg IV daily	52	22.6
	Omeprazole 20 mg po BID	33	14.3
	Omeprazole 40 mg IV BID	29	12.6
Histamin-2 Receptor Blockers	Cimetidine 200 mg IV BID	120	52.2
	Cimetidine 400 mg IV state	42	18.3
Other concomitant drugs used	NSAIDS	68	29.6
	Anticoagulant	41	17.8
	Systemic corticosteroids + NSAIDs	30	13
	systemic corticosteroids	21	9.1
	Anticoagulants + Systemic corticosteroids + NSAIDs	18	7.8
	Anticoagulants + NSAIDs	13	5.7
	Antiplatelet	10	4.3
	Anticoagulants + Antiplatelet ± Systemic corticosteroids/NSAIDs	6	2.6
	Anticoagulants + Systemic corticosteroids	5	2.2
	Anticoagulants + Antiplatelet	4	1.7
	Others*	4	1.7

NSAIDs, non-steroidal anti-inflammatory drugs; IV, intravenous; BID, two times a day. Others*, Anticoagulants + Antiplatelet + Systemic corticosteroids + NSAIDs, Antiplatelet + Systemic corticosteroids, Antiplatelet + NSAIDs, Antiplatelet + Systemic corticosteroids + NSAIDs.

### Prevalence of inappropriate use of SUP

**Figure 2:**
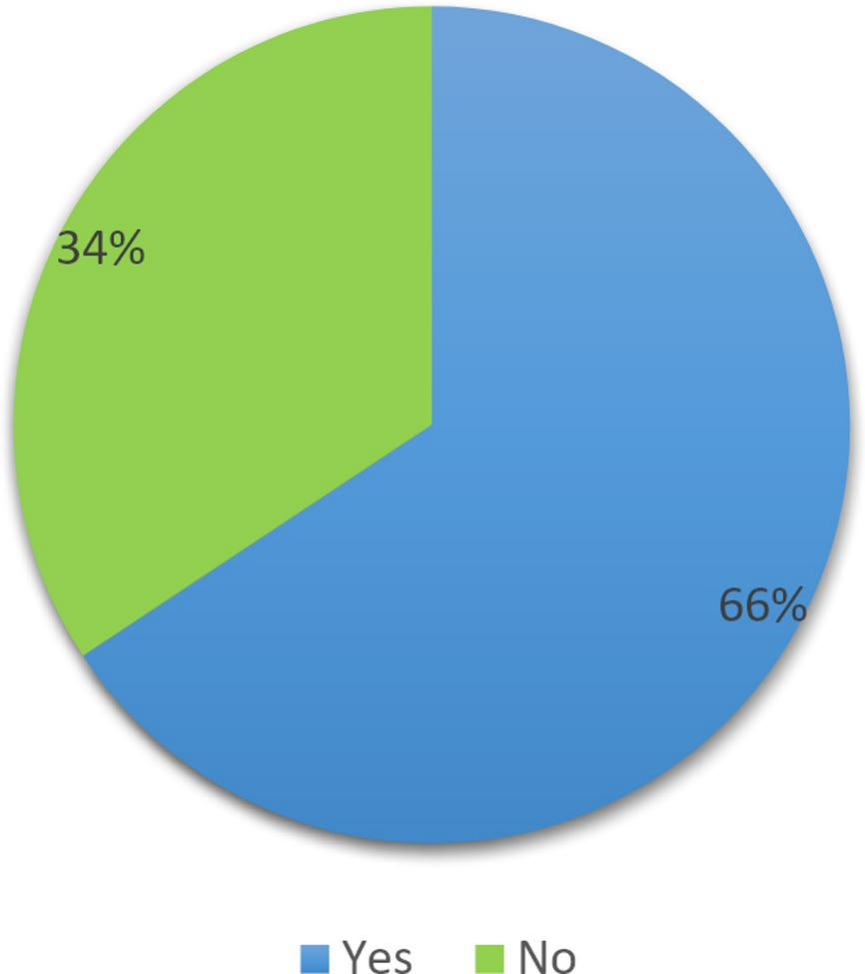
Rates of inappropriate use of SUP.

According to the ASHP Guidelines, 151 (66%) of study participants had inappropriate use of SUP (Figure 2).

### Duration of stress ulcer prophylaxis taking in days

**Figure 3:**
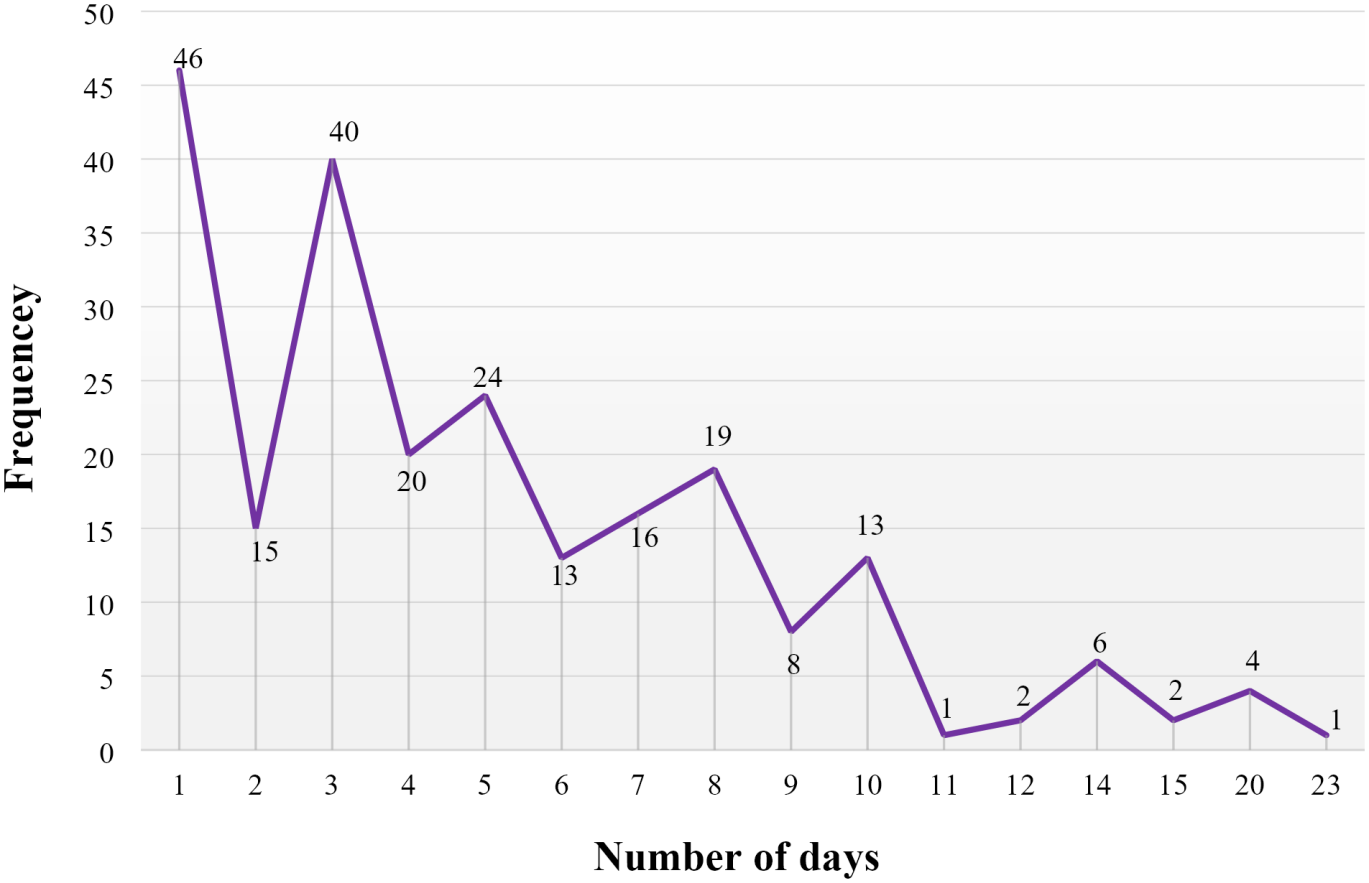
Duration of stress ulcer prophylaxis treatment, measured in days, administered in the surgical ward of DBUHGH.

Among the study participants, the mean duration of taking SUP with standard deviation was 5.18 ± 4.07 days, and the median duration of taking SUP in days was 4 days, ranging from a minimum of 1 day to a maximum of 23 days (Figure 3).

**Figure 4:**
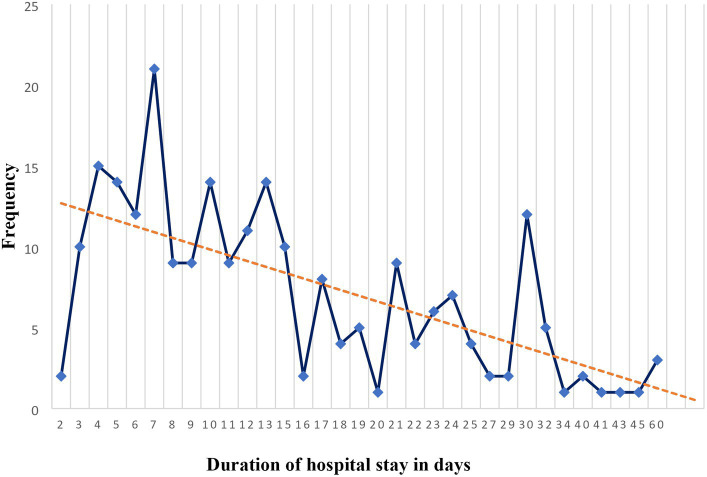
Length of hospital stay in days for patients receiving stress ulcer prophylaxis in the surgical ward of DBUHGH.

Based on the data shown in Figure 4, among the study participants who were admitted to the HGH, the median length of hospital stay for patients who received SUP was 11.5 days, with a range of 2–60 days. The mean length of hospital stay, along with its standard deviation, was 14.49 ± 10.57 days. In addition to that, the highest mode of length of hospital stay for patients who received SUP was 1 week (7 days).

### Factors associated with the inappropriate use of stress-induced ulcer prophylaxis

In univariate analysis, six of the variables studied showed an association with inappropriate use of SUP treatment. Of these candidate variables, all were categorical variables, of which five were multi-categorical variables (age, religion, Charlson Comorbidity Index score, level of SUP prescribers, and reason for taking SUP), and the remaining one variable was binary (sex). Of the six variables used for multivariate binary regression analysis, only two were identified as associated with inappropriate use of SUP treatment by multivariate binary logistic regression methods entered and cross-validated by the hierarchical regression method.

The study found that the odds of inappropriate use of SUP were 59% lower in surgical ward admitted patients whose Charlson Comorbidity Index Score was moderate (ranging from 3 to 4) (AOR = 0.41, 95% CI: 0.20–0.86, *p* = 0.02), as compared to those with mild scores ranging from 1 to 4. In addition, the odds of inappropriate use of SUP were increased 2.99-fold in patients with GI bleeding in the surgical ward (AOR =2.99, 95% CI: 1.18–7.56, *p* = 0.02), compared with patients who had not taken acid-suppressive therapy before admission (Table 5).

**Table 5:** Factors associated with the inappropriate use of stress-induced ulcer prophylaxis treatment in patients of the surgical ward of DBUHGH.

**Variables**	**Category**	**Inappropriate**		**AOR of 95% CI**	***P*-value**
		**use of SUP**			
		Yes	No		
Age	<40 years	44	65	1	0.55
	40–64 years	17	40	1.35 (0.59–3.05)	0.47
	≥65 years	30	34	0.81 (0.35–1.87)	0.63
Sex	Male	52	78	1	
	Female	39	61	0.58 (0.27–1.23)	0.16
Religion	Orthodox	80	124	1	0.15
	Muslim	10	8	0.51 (0.17–1.56)	0.24
	protestant	1	7	5.60 (0.55–56.79)	0.15
Charlson	1–2 (Mild)	43	53	1	0.08
Comorbidity	3–4 (Moderate)	6	6	0.41 (0.20–0.86)	0.02*
Index Score					
	≥5 (Severe)	1	4	0.37 (0.09–1.53)	0.17
Reason for acid-suppressive medication	On AST before admission	64	66	1	0.03*
	GI bleeding	14	41	2.99 (1.18–7.56)	0.02*
prescribing	Dyspepsia	3	1	0.248 (0.02–3.24)	0.29
	Upper GI tract bleeding	5	8	2.40 (0.61–9.34)	0.21
Level of SUP prescriber	Intern	22	25	1	0.72
	General Practitioner	47	70	0.91 (0.40–2.07)	0.82
	Specialist	22	44	1.24 (0.49–3.07)	0.65

*Indicates statistical significance. AST, acid suppression therapy; SUP, stress ulcer prophylaxis; GI, gastrointestinal; COR, crude odds ratio; AOR, adjusted odds ration.

## Discussion

In the current study, SUP was prescribed to more than two-thirds (79.1%) of surgical ward admitted patients. This is higher than the study conducted in the USA (which included 963 participant data with a retrospective chart review), which reported the use of SUP (32%) of admitted patients (28). This might be due to differences in the study setting, study participant characteristics, and level of prescribers.

The data observed in our study indicated that the mean age of study participants was 47.2 years (SD ± 20.4), and more than half, 130 (56.5%), were women. This finding is higher than the USA the mean age of the study participants was 53.2 years (±17.4) and the majority of participants were men 74 (56.9%) (35).

Our study showed that surgical ward admitted patients frequently used appropriate SUP medications. Approximately 66% of study participants did not meet SUP criteria based on ASHP guidelines, which was interpreted as inappropriate use of SUP. This finding was higher than the study reported in the USA (22%) (28), China % (1), and Gondar, Ethiopia (63.4%) (36), and lower than the study conducted in University Malaya Medical Centre Malaysia (96.4%) (37) and Jordan (86%) (38). A possible justification might be the study was performed in Malaysia in a tertiary hospital medical ward, and study participants might have had multiple co-morbidities, stayed longer periods in the hospital, and took polypharmacy, resulting in a higher rate of inappropriate prescription of SUP. Initiatives to reduce the use of improper SUPs are thus very important and urgently needed. For SUP, Ethiopia currently lacks its own set of nationally prepared clinical guidelines. As a result, a national agreement or recommendation is necessary to advise clinicians, as well as clinical and community pharmacists, on how to prescribe SUP. Onward visits and a specialized clinical pharmacist can be assigned to track the daily prescription of SUP. Fear of SU syndrome developing in patients outside of intensive care units who were not receiving SUP therapy was one of the factors that led practitioners to unnecessarily prescribe SUP (39). Clinical pharmacist intervention could significantly reduce the inappropriate utilization of acid-suppressive medications (ASMs), drug costs, and the risk of side effects (40–42).

Of the study participants, 75 (32.6%) were taking medication for SUP at discharge. This is lower than the published study done by USA (37.2%) (35). The study conducted in the USA indicated that 75% of the study participants continued on a PPI at the time of discharge (28).

Sixty-seven (29.1%) of PPIs were prescribed as SUP. This is higher than Malaysia (23.9%) (43) and lower than the study performed in Gonder, Ethiopia 76/82 (92.7%) (36), USA (70.9%) (35), Jordan 56% (38), China 96.1% (1), USA (70.0%) (44), Singapore (46.5%) (45), and Ireland (79.0%) (46). Furthermore, the literature reported that 48% (47), 61.6% (48), and 69% (49) of inpatients in the surgery department were found to be inappropriately prescribed PPIs for SUP. Based on recent published studies, PPIs seem to be more effective than H2RAs for SUP (50).

As for the type of PPIs taken for SUP, the prevalence of intravenous omeprazole 40 mg daily was 52 (22.6%). This is lower than a study in China (95.3%) (1). The study conducted in the USA indicated that 75% of the study participants took omeprazole 20 mg capsule daily as SUP (28). In our observation, omeprazole was the only prescribed PPI for SUP. The possible explanation is that, in our study setting, out of all PPIs’, only omeprazole is available in IV and PO dosage forms during the study period. However, Ethiopia does not have any PPI lists for SU prevention. According to the literature, injections administered to inpatients with nil-by-mouth situations or who encounter severe motility difficulties have been deemed suitable (49). Oral PPIs’ effectiveness was comparable to injectable formulations at comparable doses, but they were more affordable and had fewer difficulties than intravenous administration (47, 49). This highlights the need for clinical pharmacists to intervene and recommend appropriate drug delivery routes for hospital patients.

The mean duration of taking SUP was 5.18 ± 4.07 days. Based on the study conducted in China, the mean duration of SUP was 3.65 ± 3.24 days (1), which appears to be shorter than our finding in our study. However, the USA reported that most patients received SUP for a mean duration of 6.3 ± 4.5 (SD) days (47), which was longer than our finding. This might be explained by the fact that physicians did not reassess the need for PPI use regularly (48).

Study participants who have the Charlson comorbidity index score of moderate (3–4) and GI bleeding had been significantly associated with inappropriate use of SUP in surgical ward admitted patients. On the other hand, studies conducted in Lebanon (51), USA (52), and Iran (53) indicate that increasing age, being male, PPI indications not documented in the chart, and concomitant use of NSAIDs and anticoagulants were associated with inappropriate use of SUP. The possible justification is that participants who have comorbidity and GI bleeding may have an increased likelihood of receiving an incorrect SUP prescription.

## 5 Study limitations

Due to the small size of the inpatient population and the single location of this study, it was not possible to extrapolate the findings to all hospitals in Ethiopia.

Incomplete electronic records may also be another potential limitation. Patients using acid-suppressive therapy for whom there was no indication on the computerized record were assumed to be taking it as SUP.

## 6 Conclusion

In our study, inappropriate SUP use was common in the surgical ward of DBUHGH. As a result, our institution does not strictly follow the SUP criteria of the ASHP guidelines. With this finding, it is evident that specialized efforts are needed to prevent prescribing inappropriate SUP to surgical patients, and institution-specific SUP protocols are required to aid clinicians in identifying appropriate candidates for SUP.

## Data availability statement

The raw data supporting the conclusions of this article will be made available by the authors, without undue reservation.

## Ethics statement

The studies involving humans were approved by From Debre Berhan University Asrate Woldeyes Health Sciences Campus Institutional Review Board (ERB), ethical clearance of the study was obtained. The studies were conducted in accordance with the local legislation and institutional requirements. The participants provided their written informed consent to participate in this study.

## Author contributions

AT: Conceptualization, Data curation, Formal analysis, Funding acquisition, Investigation, Methodology, Project administration, Resources, Software, Supervision, Validation, Visualization, Writing – original draft, Writing – review & editing. DB: Conceptualization, Data curation, Formal analysis, Funding acquisition, Investigation, Methodology, Project administration, Resources, Software, Supervision, Validation, Visualization, Writing – original draft, Writing – review & editing. YW: Investigation, Project administration, Software, Supervision, Writing – original draft, Writing – review & editing. BE: Conceptualization, Investigation, Project administration, Software, Writing – original draft, Writing – review & editing. HH: Investigation, Methodology, Project administration, Supervision, Writing – original draft, Writing – review & editing. FG: Conceptualization, Investigation, Methodology, Project administration, Writing – original draft, Writing – review & editing. AG: Conceptualization, Formal analysis, Methodology, Supervision, Visualization, Writing – original draft, Writing – review & editing. BA: Conceptualization, Methodology, Writing – original draft, Writing – review & editing. MA: Conceptualization, Investigation, Supervision, Validation, Writing – original draft, Writing – review & editing. DT: Conceptualization, Data curation, Investigation, Methodology, Writing – original draft, Writing – review & editing. MF: Conceptualization, Data curation, Investigation, Methodology, Software, Supervision, Writing – original draft, Writing – review & editing. MG: Conceptualization, Data curation, Investigation, Methodology, Software, Supervision, Writing – original draft, Writing – review & editing. MM: Conceptualization, Investigation, Methodology, Software, Writing – original draft, Writing – review & editing. TD: Conceptualization, Data curation, Formal analysis, Investigation, Methodology, Resources, Writing – original draft. SA: Writing – review & editing, Writing – original draft, Visualization, Validation, Supervision, Software, Resources, Project administration, Methodology, Conceptualization, Data curation, Formal analysis, Funding acquisition, Investigation.
